# Long-lasting responses after discontinuation of nivolumab treatment for reasons other than tumor progression in patients with previously treated, advanced non-small cell lung cancer

**DOI:** 10.1186/s40880-019-0423-3

**Published:** 2019-11-21

**Authors:** Hideharu Kimura, Tomoyuki Araya, Taro Yoneda, Hiroki Shirasaki, Koji Kurokawa, Tamami Sakai, Hayato Koba, Yuichi Tambo, Shingo Nishikawa, Takashi Sone, Kazuo Kasahara

**Affiliations:** 1 Respiratory Medicine, Kanazawa University Hospital, Takara-machi 13-1, Kanazawa, Ishikawa 920-8641 Japan; 2 Respiratory Medicine, National Hospital Organization Kanazawa Medical Center, Kanazawa, Ishikawa 920-8650 Japan; 3 Respiratory Medicine, Komatsu Municipal Hospital, Komatsu, Ishikawa 923-8560 Japan; 4 Respiratory Medicine, Fukui-ken Saiseikai Hospital, Fukui, Fukui 918-8503 Japan; 5 Respiratory Medicine, Ishikawa Prefectural Central Hospital, Kanazawa, Ishikawa 920-8530 Japan; 6 Internal Medicine, Keiju Medical Center, Nanao, Ishikawa 926-8605 Japan; 7 Regional Respiratory Symptomatology, Kanazawa University Graduate School of Medical Science, Kanazawa, Ishikawa 920-8641 Japan

Dear Editor,

Nivolumab, an inhibitor of programmed cell death 1 (PD-1), is an immune checkpoint inhibitor (ICI) that enhances T cell functions by preventing negative regulation of cancer immunity, and it has shown clinically significant efficacy and tolerability in various types of cancer. Based on the results of randomized phase III trials comparing nivolumab with docetaxel [1, 2], nivolumab is now used in clinical practice for patients with previously treated advanced non-small cell lung cancer (NSCLC). Furthermore, the most recent Japan Lung Cancer Society guidelines include nivolumab monotherapy in the systemic treatment strategy for previously treated, locally advanced or metastatic NSCLC [3]. However, ICIs induce characteristic immune-related adverse events (irAEs), which are not seen with conventional cytotoxic agents or molecular targeted agents. IrAEs can occur in any organ system, most typically the skin, lung, and gastrointestinal, hepatic, and endocrine systems. Based on the results of two randomized phase III trials, grade 3–4 adverse events (AEs) developed in 7% [1] and 10% [2] of patients in the nivolumab groups, respectively, but no grade 5 events were seen. In the nivolumab group, 3% and 5% of patients discontinued nivolumab due to AEs, and the rate of drug discontinuation was lower than that in the docetaxel group [1, 2]. We reported a case of advanced lung squamous cell carcinoma that showed long-lasting tumor shrinkage after discontinuation of nivolumab treatment under no further cancer treatments [4]. For that patient, we had no choice but to discontinue nivolumab treatment due to onset of interstitial lung disease, despite a good response to the first two nivolumab doses. Continued tumor shrinkage under no further treatments has not been seen in patients receiving other antitumor agents and may be a unique feature of nivolumab treatment, and potentially other ICIs. To expand on these observations, we examined the clinical characteristics of patients with advanced NSCLC who received nivolumab treatment but discontinued it for a reason other than tumor progression.

This retrospective observational study was performed to obtain real-world data on the prognosis of patients who discontinued nivolumab (240 mg, intravenous drip infusion, every 2 weeks), treatment but showed no progression. All patients with advanced NSCLC who had received nivolumab monotherapy and discontinued it by 31 March 2016 were initially selected from each institution. Of these 124 patients, 17 who had discontinued nivolumab due to reasons other than disease progression were included in the analysis (Table 1). This study was initially approved by Kanazawa University (approval no. 2423-2) and subsequently approved by the other five institutions. We collected limited and anonymized clinical data, and no additional interventions were performed. Therefore, written informed consent was not required.

**Table 1:** Characteristics of patients with advanced NSCLC who received nivolumab monotherapy but discontinued it for a reason other than tumor progression

Patient No.	Sex	Histological type	*EGFR* mutation	*ALK* fusion gene	Treatment regimen prior to nivolumab	Nivolumab treatment				
						Age at initiation (years)	ECOG PS at initiation	Number of doses	Response^a^	Reason for discontinuation^b^
1	F	Adenocarcinoma	wt	wt	PEM + BEV	79	1	1	NE	Bone fraction
2	M	Pleomorphic	wt	wt	CBDCA + PTX + BEV, DTX	60	1	3	PR	irAE (grade 5 encephalitis)
3	M	Adenocarcinoma	wt	wt	CBDCA + PEM	61	1	6	SD	Rejection
4	M	Squamous	wt	wt	CBDCA + PTX, VNR, CBDCA + S-1	68	0	2	PR	irAE (grade 2 pneumonitis)
5	M	NOS	wt	wt	CBDCA + PTX + TRT, PEM + BEV	55	2	1	NE	irAE (grade 2 pneumonitis)
6	M	Squamous	wt	wt	CBDCA + PTX	78	1	1	NE	irAE (grade 2 pneumonitis)
7	M	Adenosquamous	wt	wt	GEM + VNR, PEM, DTX, PEM + BEV	90	1	1	SD	Infection
8	M	Adenocarcinoma	wt	wt	CBDCA + PTX + TRT, CBDCA + PEM, DTX	75	1	31	SD	irAE (grade 2 pneumonitis)
9	M	Squamous	wt	wt	CDDP + S-1, CBDCA + nabPTX	67	1	2	PR	irAE (grade 3 dermatitis)
10	M	Adenosquamous	wt	wt	TRT, DTX	83	1	11	PR	Heart failure
11	F	Adenocarcinoma	wt	wt	PEM, DTX	79	1	8	SD	irAE (grade 3 arthritis)
12	M	Squamous	wt	wt	CBDCA + nabPTX	79	2	3	SD	irAE (grade 1 pneumonitis)
13	M	Adenocarcinoma	wt	wt	CBDCA + PEM	69	2	2	SD	irAE (grade 2 pneumonitis)
14	M	Adenocarcinoma	L858R	wt	CBDCA + PEM, Afatinib, DTX, Erlotinib + BEV, nabPTX, GEM, VNR	64	1	1	NE	irAE (grade 5 pneumonitis)
15	M	Adenocarcinoma	NE	NE	CDDP + PEM	59	1	1	NE	irAE (grade 5 pneumonitis)
16	F	Squamous	wt	wt	CBDCA + S-1	76	0	2	NE	irAE (grade 3 pneumonitis)
17	M	Squamous	NE	NE	TRT, CBDCA + S-1, VNR	68	2	5	SD	Rejection

*NSCLC* non-small cell lung cancer, *EGFR* epidermal growth factor receptor, *ALK* anaplastic lymphoma kinase, *ECOG PS* the Eastern Cooperative Oncology Group performance status, *F* female, *M* male, *NOS* not otherwise specified, *wt.* wild type, *NE* not evaluable, *PEM* pemetrexed, *BEV* bevacizumab, *CBDCA* carboplatin, *PTX* paclitaxel, *DTX* docetaxel, *VNR* vinorelbine, *TRT* thoracic radiotherapy, *GEM* gemcitabine, *CDDP* cisplatin, *nabPTX* nab-paclitaxel, *PR* partial response, *SD* stable disease, *irAE* immune-related adverse event

^a^The responses were classified by the Response Evaluation Criteria in Solid Tumors 1.1

^b^Adverse events were graded according to the Common Terminology Criteria for Adverse Events (CTCAE) version 5.0

Progression-free survival (PFS) was defined as the duration from the initiation of nivolumab treatment to disease progression or death. The PFS of the patients ranged from 13 to 580 days, with a median PFS of 163 days (Fig. 1). Notably, 6 of the 17 patients (patients #3, 4, 9, 11, 13, and 16) had a long PFS (≥ 6 months) with no additional treatment after nivolumab treatment for NSCLC. For these 6 patients, nivolumab treatment was discontinued because of irAEs in 5 and refusal to continue treatment in 1 patient. Of the 6 patients, 4 were male, 3 had adenocarcinoma, and 3 had squamous cell carcinoma; none had *EGFR* mutations or anaplastic lymphoma kinase (*ALK*) fusion genes; the age at treatment initiation ranged from 61 to 79 years, and the Eastern Cooperative Oncology Group (ECOG) performance status (PS) was good (Table 1). The response rates were 33.3% among patients with a long PFS and 18.2% among patients with a short PFS. Two patients died within 1 month after the initiation of nivolumab treatment: patient #14 died on day 13 and patient #15 on day 28. In both patients, the cause of death was grade 5 pneumonitis induced by nivolumab. Other antitumor agents were administered in only 2 patients after tumor progression. According to Kaplan–Meier analyses of PFS, patients with a good PS or irAEs had a longer PFS than their counterparts, although without significant differences (Additional file 1: Figure S1). However, the sample size was too small to conclude any overall trends.

**Fig. 1:**
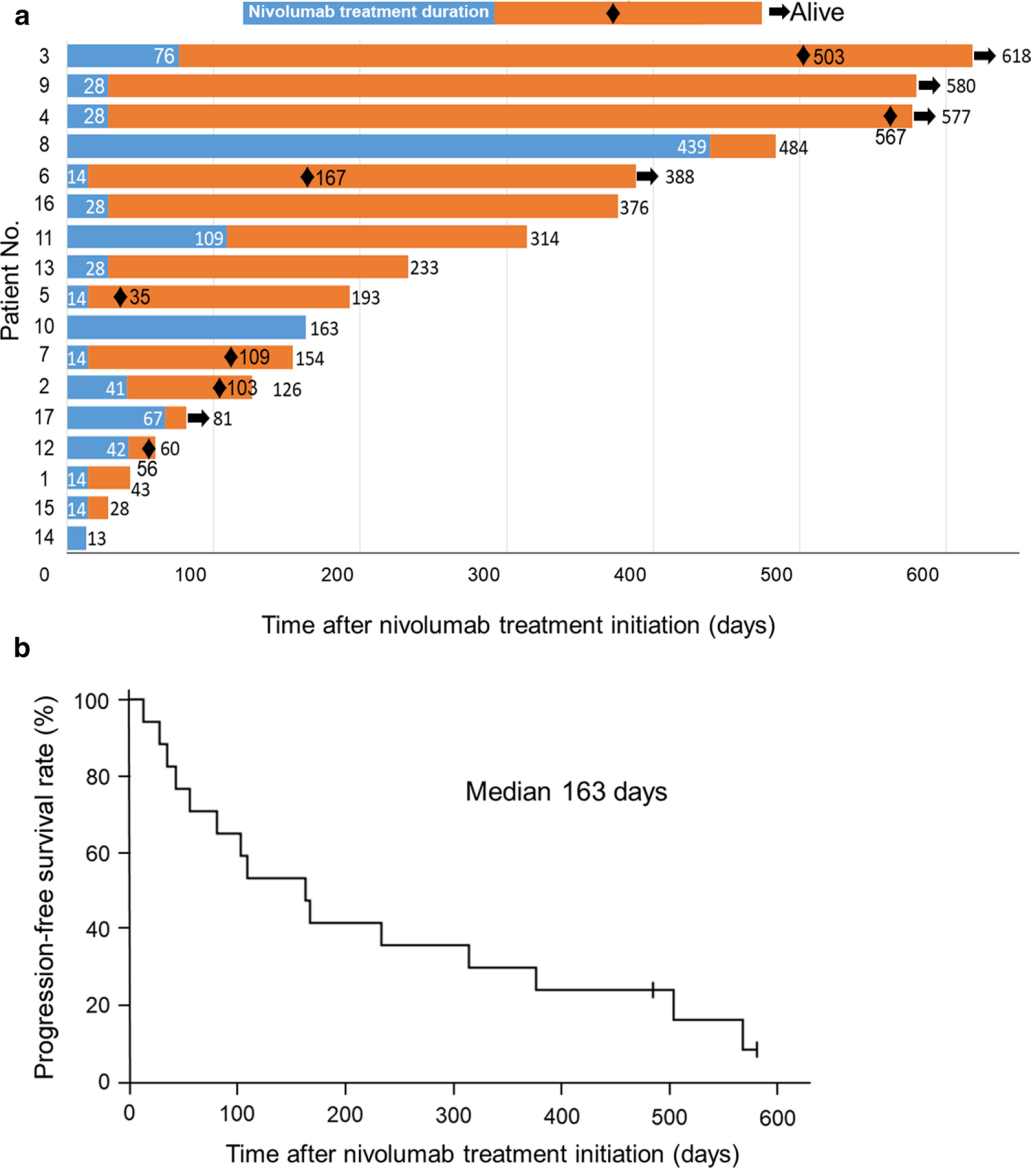
Kaplan–Meier survival analysis of the 17 patients with advanced non-small cell lung cancer who had received nivolumab and discontinued it due to reasons other than disease progressions. **a** The duration of nivolumab treatment and survival after treatment discontinuation. The blue bars indicate the duration of nivolumab treatment, and orange bars indicate the survival after the discontinuation of nivolumab treatment. Black diamonds indicate the time of tumor progression, and arrows indicate the alive patients by the last follow-up. Patient numbers at the vertical axis heading correspond to those in the first column of Table 1. **b** Progression-free survival curve of the 17 patients

Two randomized phase III trials (CheckMate 017 for patients with squamous NSCLC and CheckMate 057 for patients with non-squamous NSCLC) evaluated the survival benefits of nivolumab versus docetaxel in previously treated patients with advanced NSCLC [5]. One characteristic of PD-1 inhibitors is that some patients have the expectation of long-term survival compared with conventional cytotoxic chemotherapy. The 2-year PFS rates of the patients treated with nivolumab were 16% and 12% (in CheckMate 017 and 057, respectively). By comparison, the 2-year PFS rate of the patients receiving docetaxel treatment was only 1% in the CheckMate 057, and that in CheckMate 017 was not calculated because no docetaxel-treated patients were followed up for 2 years. Interestingly, 6 of the patients who received nivolumab treatment (1 in CheckMate 017 and 5 in CheckMate 057) achieved a long-lasting response (> 6 months) under no additional treatment. The predictors for long-term survival after nivolumab treatment have been analyzed but remained unclear.

The CheckMate 153 trial compared patients who discontinued nivolumab within 1 year with those who continued treatment until disease progression or severe AEs, and the major conclusion was that survival was significantly longer in the continuation group than in the discontinuation group [6]. However, we focused on the long-term survivors after nivolumab discontinuation in the present study. The 1-year PFS rate of the patients in the discontinuation group was approximately 40%.

A key finding of the present study was that some patients had a long survival after nivolumab discontinuation despite no further anti-cancer treatment. We speculate that patients who discontinue treatment due to AEs, especially irAEs, will have long-lasting responses to nivolumab. Haratani et al. [7] evaluated the relationship between irAEs and nivolumab efficacy in patients with advanced NSCLC treated with nivolumab in the second-line setting or later. The patients with irAEs had a higher response rate and longer PFS and overall survival compared with those without irAEs [7]. Meanwhile, there were two early (< 1 month) deaths related to irAEs. The mechanism explaining why the patients with irAEs had longer survival compared with those without irAEs remains unknown. One possibility is that tumor-specific T cells also recognize antigens expressed on normal cells, thereby inducing irAEs. Many commonly targeted tumor antigens are also expressed in normal tissues [8]. Hasan Ali et al. [9] showed that the pattern of lymphocytic skin infiltration in patients with skin toxicity differed according to the histological subtype of NSCLC and was associated with the response to nivolumab. Although only one patient discontinued nivolumab due to skin toxicity in the present study, other irAEs may involve similar lymphocytic infiltrations.

Our results should be confirmed by a larger-scale study. The present study was retrospective, and the results were influenced by the decisions of physicians, such as the time points of nivolumab discontinuation and initiation of subsequent treatments. In other words, we obtained data reflecting actual clinical settings. In a phase III trial that compared pembrolizumab, a PD-1 inhibitor, with chemotherapy in patients with untreated advanced NSCLC expressing programmed death-1 ligand-1 (PD-L1) in at least 50% of tumor cells, pembrolizumab resulted in longer PFS and overall survival than did chemotherapy [10]. Since that trial, immunohistochemical analysis of PD-L1 expression is commonly performed in clinical settings to decide whether pembrolizumab monotherapy should be used as the first-line treatment. Unfortunately, PD-L1 expression was not investigated in the patients of the present study because nivolumab treatment was initiated before approval of the PD-L1 immunohistochemistry test.

In conclusion, some patients with previously treated, advanced NSCLC who discontinued nivolumab treatment for reasons other than tumor progression may have potential for a long-lasting treatment response, and irAE onset and a good ECOG PS at nivolumab initiation may be predictive markers of a long-lasting treatment response.

## Supplementary information

**Additional file 1: Figure S1.** Kaplan–Meier progression-free survival curves of the 17 patients stratified by irAE and PS. (A) Survival curves of patients with or without irAE. (B) Survival curves of patients with good or poor PS before nivolumab treatment.

## Data Availability

The datasets used and/or analyzed during the report are available from the corresponding author on reasonable request.

## Abbreviations

NSCLC: non-small cell lung cancer
irAE: immune-related adverse event
PFS: progression-free survival
ECOG: the Eastern Cooperative Oncology Group
PS: performance status
EGFR: epidermal growth factor receptor
ALK: anaplastic lymphoma kinase
PD-1: programmed cell death 1
ICI: immune checkpoint inhibitor
PD-L1: programmed death-1 ligand-1
