# Supplementary figures and images for: Long-lasting responses after discontinuation of nivolumab treatment for reasons other than tumor progression in patients with previously treated, advanced non-small cell lung cancer
Source: Cancer Commun (Lond). 2019 Nov 21;39:78. doi: 10.1186/s40880-019-0423-3 (PMC6873691; doi:10.1186/s40880-019-0423-3)

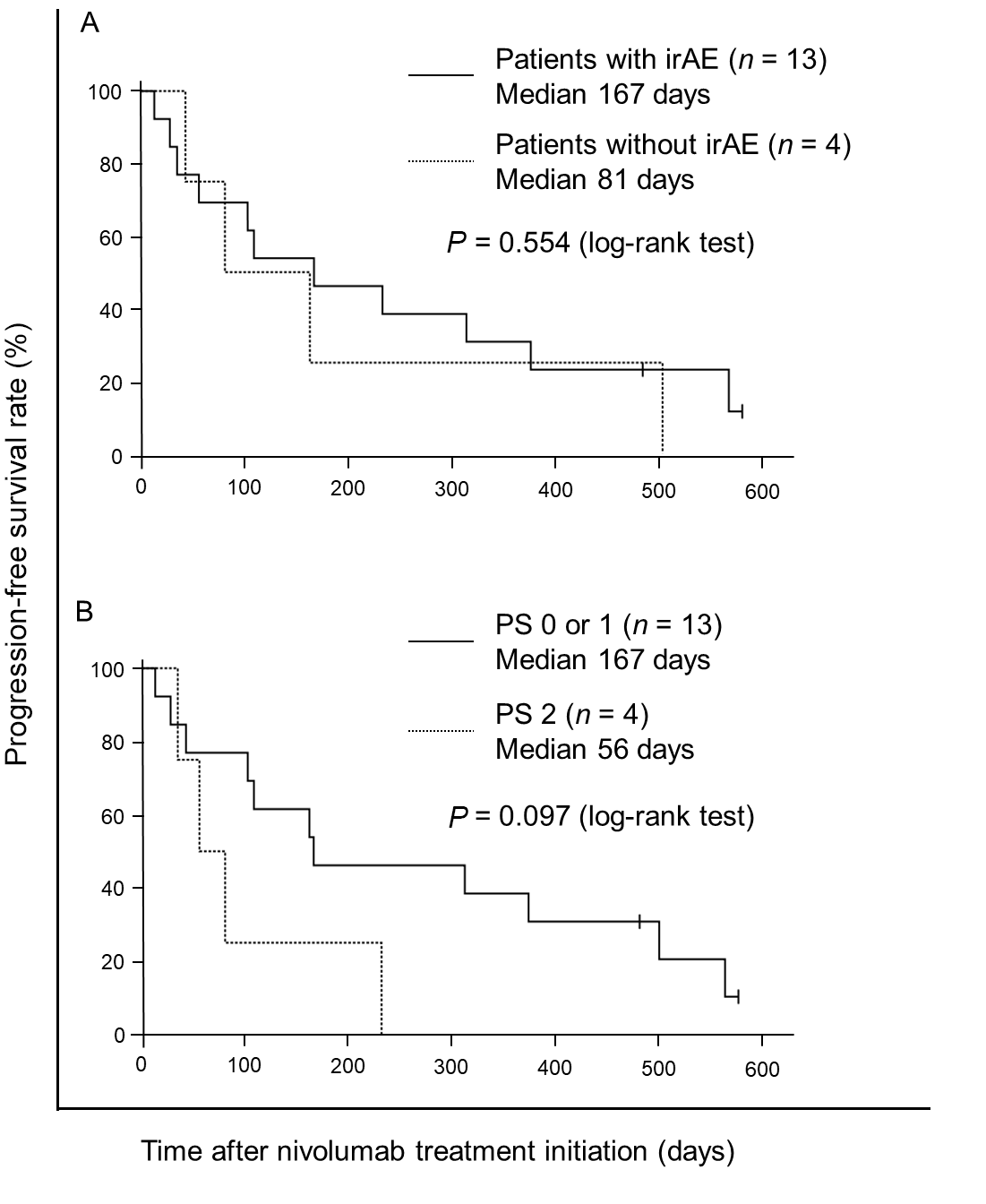

Supplement: Supplementary file 1 — Additional file 1: Figure S1. Kaplan–Meier progression-free survival curves of the 17 patients stratified by irAE and PS. (A) Survival curves of patients with or without irAE. (B) Survival curves of patients with good or poor PS before nivolumab treatment. [file 40880_2019_423_MOESM1_ESM.tif]
